# Effects of variable-temperature drying on nutritional components, antioxidant capacity, and flavor (volatile and non-volatile compounds) of Xinjiang figs

**DOI:** 10.1016/j.fochx.2026.104439

**Published:** 2026-09-12

**Authors:** Yaxuan Liao, Liqing Yang, Hao Dong, Weida Zhang, Lingling Li, Minrui Guo, Wanting Yang, Guogang Chen

**Affiliations:** a College of Food Science and Technology, Shihezi University, Shihezi 832000, China; b Xinjiang Shihezi Vocational Technical College, Shihezi, Xinjiang 832000, China; c Key Laboratory of Characteristics Agricultural Product Processing and Quality Control (Co-construction by Ministry and Province) Ministry of Agriculture and Rural Affairs, School of Food Science and Technology, Shihezi University, Shihezi, Xinjiang 832000, China; d Key Laboratory for Food Nutrition and Safety Control of Xinjiang Production and Construction Corps, School of Food Science and Technology, Shihezi University, Shihezi, Xinjiang 832000, China; e Shihezi Testing Institute of Quality and Metrology, Shihezi 832000, PR China

**Keywords:** Variable-temperature drying, *Ficus carica* L., Energy consumption, Nutrient retention, Volatile profile

## Abstract

A two-stage variable-temperature drying (VTD) process was optimized to improve the drying efficiency and quality of Xinjiang figs. The optimal conditions were 55 °C followed by 80 °C at a loading capacity of 2.73 g/dm^2^. Compared with constant-temperature drying at 60 °C, VTD shortened the drying time by 44.8%, reduced specific energy consumption by 20.4%, and decreased the browning degree by 22.8%. VTD also increased the retention of total phenolics, total flavonoids, and ascorbic acid by 17.59%, 27.78%, and 43.29%, respectively, resulting in higher antioxidant capacity. The summed semi-quantitative volatile abundance was higher in VTD than in CTD (39.78 versus 7.01 μg/g DW), mainly due to benzaldehyde and selected alcohols. Overall, two-stage VTD improved drying efficiency, nutrient retention, antioxidant capacity, and the volatile profile of dried figs, indicating its potential for further development of dried-fig processing under the tested conditions.

## Highlights

• Variable-temperature drying (VTD) reduces time by 44.8% and energy by 20.4%.
• VTD improves retention of phenolics, flavonoids, and ascorbic acid.
• VTD preserves fig microstructure and is associated with lower browning.
• VTD alters volatile profiles consistent with putative thermal pathways.
• VT-E volatiles increase mainly because of benzaldehyde accumulation.

## Graphical abstract

**Figure:**
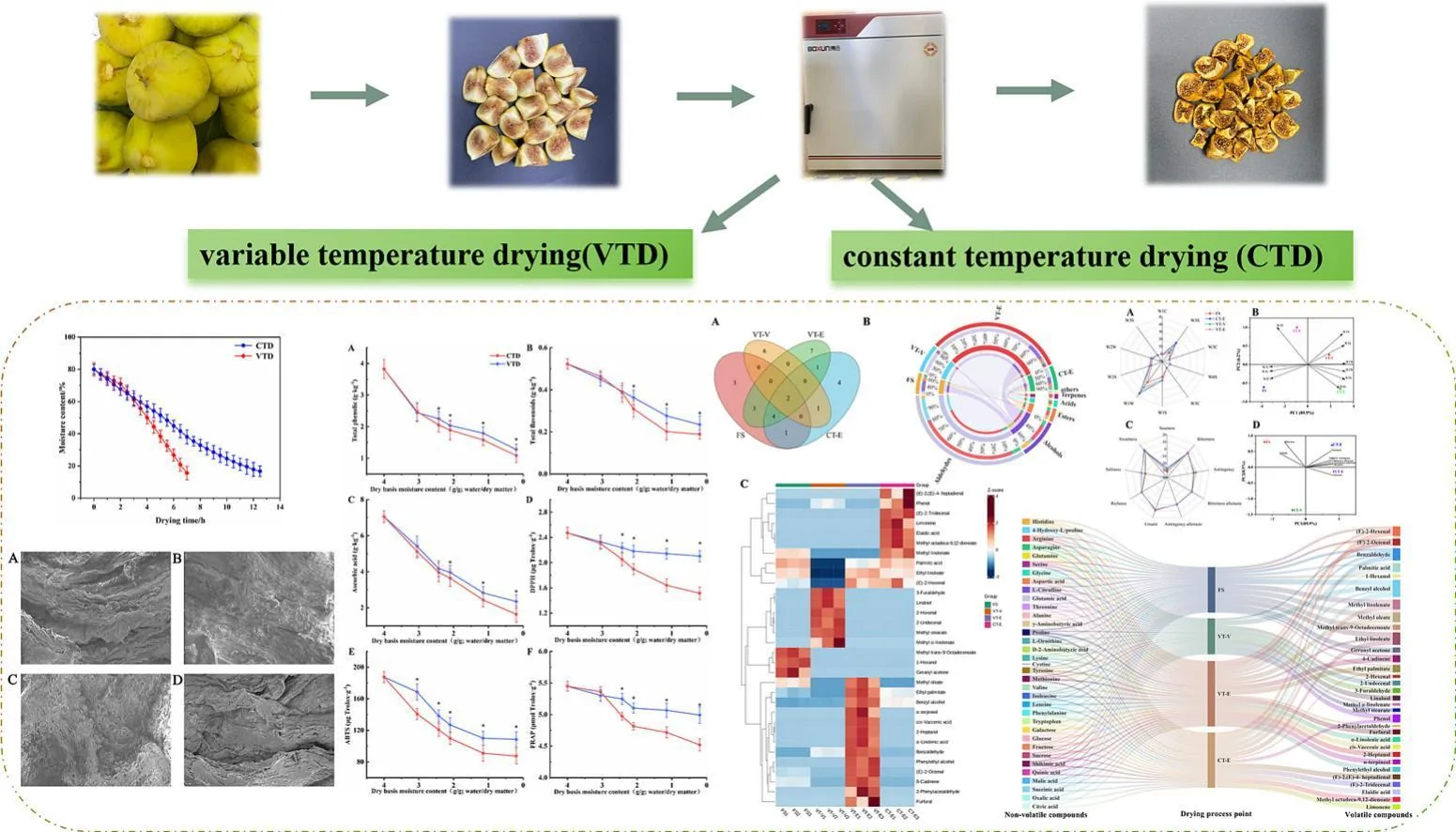

## 1 Introduction

Fig (*Ficus carica* L.) is highly valued worldwide for its remarkable antioxidant, anti-inflammatory, and health-promoting properties, attributed to its abundance of bioactive compounds such as polyphenols, flavonoids, and dietary fiber (Ali et al., 2025; Russo et al., 2014; Tahir et al., 2024). In China, figs are cultivated in regions including Shandong, Xinjiang, and Jiangsu. Among these, Kashgar in Xinjiang has become the primary production area due to its superior light and heat resources and geographical advantages. However, factors such as high moisture content, thin skin, and underdeveloped epidermal protective tissues render figs highly perishable (Jafari et al., 2025), with quality deterioration occurring within just 7–10 days of cold storage. Issues related to storability and transport distance severely constrain the development of the fig industry, with over 90% of figs being distributed in the form of dried products (Mat Desa et al., 2019).

Currently, constant-temperature drying (CTD) is widely employed in industrial production for preparing dried figs. Nevertheless, the dense skin and natural wax layer of whole figs create substantial resistance to moisture diffusion, resulting in prolonged drying cycles and impeded internal moisture removal. Although pretreatment by cutting increases the evaporation surface area, constant high temperatures can easily induce case-hardening—where rapid surface dehydration forms a moisture barrier that hinders internal water migration. This not only increases energy consumption but also exacerbates enzymatic and non-enzymatic browning, as well as the degradation of heat-sensitive nutrients (Liu et al., 2022). In contrast, variable-temperature drying (VTD), as a dynamic temperature control technology, allows staged temperature adjustments based on the moisture migration behavior and quality evolution characteristics of the material (Liao et al., 2024). Existing studies have confirmed that VTD outperforms CTD in preserving the physical properties, such as color, texture, and rehydration capacity, and flavor of fruits such as jujube (Liao et al., 2024), Chinese yam (Li et al., 2023), and Barton pecan (Roberto Thewes et al., 2022). These studies have mainly focused on drying kinetics, color, texture, energy consumption, or selected volatile compounds. However, the effects of staged temperature regulation on the combined evolution of nutrient-related compounds, antioxidant capacity, soluble sugars, organic acids, free amino acids, and volatile profiles in figs remain insufficiently characterized. In particular, limited information is available regarding how the compositional changes occurring before and after the temperature transition are statistically associated with the final volatile profile of dried figs.

Therefore, this study aimed to: (1) optimize the initial temperature, final temperature, and loading density of a two-stage VTD process using browning degree and specific energy consumption as process-level responses; (2) compare the physical properties, nutrient retention, antioxidant capacity, and volatile and non-volatile profiles of figs subjected to optimized VTD and CTD at 60 °C; and (3) explore statistical associations between non-volatile precursors and volatile compounds at different drying stages. The novelty of this work lies in the stage-resolved and multidimensional characterization of Xinjiang figs during two-stage VTD rather than in the direct verification of specific chemical reaction pathways.

## 2 Materials and methods

### 2.1 Materials

Xinjiang Zaohuang figs (*Ficus carica L*.) produced from Kashgar, Xinjiang, were used in this study. The fresh figs had a wet basis moisture content of approximately 80%. The figs were sent to the laboratory of the Food College of Shihezi University immediately after picking. Samples that were free of damage, decay, and of uniform size were selected and stored at a temperature of (0 ± 1) °C and a relative humidity of 80 to 95% for the drying experiments.

### 2.2 Drying experiments and process optimization

200 g figs of uniform color, size, and shape were selected, cut into 6 evenly sized petals, and laid flat in a drying oven (BGZ246, Shanghai Boxun Medical Biological Instrument Corp., Shanghai, China). The oven was preheated for 30 min before the samples were added. Based on the results of preliminary single-factor experiments, the VTD parameters were systematically optimized. Specifically, the effects of pre-temperature (45, 50, 55, 60, and 65 °C), moisture conversion point (40, 50, 60, 70, and 80%), post-temperature (70, 75, 80, 85, and 90 °C), air velocity (6, 7, 8, 9, and 10 m/s), and loading capacity (0.50, 1.25, 2.00, 2.75, and 3.50 g/dm^2^) on drying performance were evaluated in five independent one-factor-at-a-time experiments, with each factor tested at five levels. Two-stage temperature control is simpler to implement at industrial scale and imposes lower operational complexity than multi-stage profiles. Therefore, the two-stage configuration was adopted for further optimization. Based on the single-factor results, the moisture conversion point (fixed at 60%) and air velocity (fixed at 7 m/s) were excluded from further optimization, as these factors showed no significant effect on browning degree or specific energy consumption (SEC). Subsequently, a three-factor, three-level Box–Behnken response surface design was conducted using pre-temperature (A: 50, 55, 60 °C), post-temperature (B: 75, 80, 85 °C), and loading capacity (C: 2.00, 2.75, 3.50 g/dm^2^) as independent variables, with browning degree (Y1) and SEC (Y2) as response variables. The VTD process was implemented as follows: samples were first dried at the pre-temperature until the moisture content decreased to the predetermined conversion point (60% wet basis), after which the temperature was switched to the post-temperature and maintained until the final moisture content reached 17% (wet basis), as stipulated by the national standard (GH/T 1364–2021) for dried figs (moisture content <20%). Moisture content was monitored by periodic weighing of the samples at 30-min intervals throughout the drying process to ensure accurate temperature switching. The optimal VTD conditions obtained from the response surface model were pre-temperature = 55 °C, post-temperature = 80 °C, loading capacity = 2.73 g/dm^2^, and these conditions were used for all subsequent VTD experiments.

Browning degree and SEC were selected as optimization responses because they represented two directly measurable and operationally relevant objectives: product appearance and process energy demand. Nutritional, antioxidant, texture, and volatile attributes were subsequently evaluated using samples produced under the optimized process. They were not included in the Box–Behnken design because comprehensive chemical analysis of every experimental run was beyond the predefined scope of process optimization. Consequently, the optimized conditions should be interpreted as optimal with respect to browning degree and SEC, rather than as a global optimum for all nutritional and flavor-related attributes. At the same time, samples dried at a constant temperature of 60 °C were used as controls for subsequent quality comparisons. The pre-experimental results for the sample dried at constant temperature of 60 °C yielded a browning degree of 0.57, an SEC of 5.24 kWh/kg (equivalent to 8.32 kWh/kg water removed), and a drying endpoint moisture content of 17%.

### 2.3 Determination of drying curves and energy consumption

Determination of energy consumption: The energy consumption for drying 1 kg of fresh figs was calculated according to Eq. (1), where SEC is the specific energy consumption (kWh/kg); E is the energy consumption of the meter, kWh; and M is the weight of the fresh figs, kg.

(1) SEC=E/M

Moisture content (*Mc*): *Mc* was calculated with reference to Eq. (2) (Chen et al., 2020). Where *M*_*t*_ is the mass of the figs at time t, g; *M*_*0*_ is the initial mass of the figs, g; *W*_*0*_ is the initial wet basis moisture content of the figs, %; and *Mc* is the wet basis moisture content of figs at time t, %.

(2) $$\mathrm{Mc}=\frac{M_{t}-M_{0}\left(1-W_{0}\right)}{M_{t}}$$

### 2.4 Surface color, texture analysis and microstructure

#### 2.4.1 Surface color

A colorimeter (YS3060, Shenzhen Sanensi Technology Co., Ltd., Shenzhen, China) was used to determine the color changes of the fig (Liao et al., 2025). *ΔE* represents color difference and is calculated using the following Eq. (3):

(3) $$∆E=\sqrt{{\left(L^{*}-L^{0}\right)}^{2}+{\left(a^{*}-a^{0}\right)}^{2}+{\left(b^{*}-b^{0}\right)}^{2}}\;$$

where *L*^*⁎*^, *a*^*⁎*^, and *b*^*⁎*^ are the brightness, red-green value, and blue-yellow values of figs after drying; and *L*^*0*^, *a*^*0*^, and *b*^*0*^ are the brightness, red-green, and blue-yellow values of figs before drying.

#### 2.4.2 Texture characteristics

The texture characteristics were measured using a texture analyzer (Surface Measurement Systems Ltd., UK) (Liao et al., 2025) equipped with a round 36-mm-diameter SMS P/75R probe. The compression and upward velocity were both 5 mm/s, the compression interval was 5 s, the sample deformation was 50%, and the hardness (g), adhesiveness (N·mm), gumminess (g), chewiness (g), springiness and cohesiveness were measured. Each test was performed three times.

#### 2.4.3 Microstructure analysis

The internal microstructure of the dried samples at 50× and 300× was observed using a scanning electron microscope (S-3400 N, Hitachi Ltd., Japan) according to a previously published method (Liao et al., 2025). Figs cut into 1 cm × 1 cm × 0.3 cm and fixed in glutaraldehyde at 4 °C for 10 h. Then, the samples were then dehydrated by gradient ethanol solutions (volume fractions: 30%, 50%, 70%, 90%, and 100%) for 15 min. Finally, freeze-dried to obtain SEM samples. Prior to observation, the dehydrated samples were coated with gold-palladium in an ion sputter coater.

### 2.5 Antioxidant capacity evaluation

#### 2.5.1 Determination of total phenolics, flavonoids, and ascorbic acid

Total phenolics, total flavonoids, and ascorbic acid were determined according to Liu et al. (2022) with minor modifications. Dried samples (5.0 g) were ultrasonically extracted with 80% methanol-water, and the combined supernatants were pooled to 50 mL for analysis. TPC was measured at 760 nm using the Folin-Ciocalteu method (gallic acid equivalent), and TFC was measured at 506 nm using the NaNO_2_-AlCl_3_ method (rutin equivalent). For AsA, the extract was analyzed by HPLC on a C18 column with a phosphate-methanol (90,10, *v*/v) mobile phase and detected at 254 nm against an external standard. All results are expressed on a dry matter basis (g/kg DM).

#### 2.5.2 Determination of antioxidant capacity

DPPH radical-scavenging activity, ABTS radical-scavenging activity, and FRAP (total antioxidant capacity) were determined using commercial assay kits (catalog Nos. G0128W, G0127W, and G0115W48, respectively; Suzhou Grace Biotechnology Co., Ltd., China). Briefly, for tissue samples, approximately 0.1 g of fresh sample was homogenized with 1 mL of 80% methanol (for DPPH and ABTS assays) or 80% ethanol (for FRAP assay), followed by ultrasonic extraction at 60 °C and 200–300 W for 30 min with intermittent mixing every 5 min. The extracts were centrifuged at 12,000 rpm for 10 min, and the supernatants were collected for analysis. Absorbance was measured at 517 nm for DPPH, 734 nm for ABTS, and 590 nm for FRAP. Calibration was conducted using Trolox as the standard over the range of 0–2.0 μmol for FRAP (y = 0.0972x + 0.0042), clearance rates ranging from 0% to 100% for ABTS (y = 0.5042x − 1.4213) and DPPH (y = 2.8486x + 0.7084). Reagent blanks and sample blanks were included in each assay, and each independent sample was measured in triplicate. Full independent in-house validation of the commercial-kit methods was not performed; all assays were conducted strictly according to the manufacturer's instructions. Antioxidant capacity was expressed as μg Trolox/g DW.

### 2.6 Volatile and non-volatile compounds analysis

#### 2.6.1 Soluble sugars

Following a previously published method (Liao et al., 2025), a 100 mg sample was weighed into a 2.0-mL centrifuge tube and extracted with 700 μL of 80% ethanol for 2 h. The supernatant was centrifuged at 10,000*g* for 3 min and then aspirated for analysis. A Thermo ICS5000 ion chromatography system (ICS5000, Thermo Fisher Scientific, USA) fitted with a CarboPac™ PA1 (250 × 4.0 mm) column was used to determine the monosaccharide fractions. The mobile phases were deionized water; the injection volume was 10 μL, the flow rate was 1.0 mL/min, and the column temperature was 30 °C. The results are expressed in mg/g dry weight (DW).

#### 2.6.2 Organic acids

Organic acids were determined according to a previously published method (Liao et al., 2024). Briefly, 0.1–0.5 g of sample was extracted with 300 μL methanol/chloroform (7,3) on ice for 30 min. Then, 200 μL of H_2_O was added, the mixture was centrifuged for 10 min, and the supernatant was taken for analysis. This H_2_O extraction step was then repeated. Ultra-performance liquid chromatography (UPLC; Vanquish, Thermo, USA) and high-resolution mass spectrometry (Q Exactive, Thermo, USA) were employed with the aid of an autosampler at 4 °C. The chromatographic column used was a Waters BEH C18 (50 × 2.1 mm, 1.8 μm). The mobile phase A was acetonitrile/ water (84,16, *v*/v); mobile phase B was 10 mM ammonium formate; the flow rate was 0.35 mL/min; the column temperature was 40 °C; and the injection volume was 2 μL. The results are expressed as mg/g DW.

#### 2.6.3 Free amino acids

A 1 g sample was extracted with 50 mL hydrochloric acid (0.1 mol/L) for 45 min at 25 °C followed by centrifugation at 8000*g* for 10 min. The supernatant was filtered, and analysis was performed using Biochrom Ltd. (UK). The results are expressed as mg/g DW.

#### 2.6.4 Aroma-compound extraction and analysis

HS-SPME-GC-MS was used to detect volatile components according to a previously published method (Liu et al., 2022). First, 0.1 g of the sample was transferred to a 15 mL extraction bottle containing 5 mL ultrapure water and 10 μL of a 4-methyl-2-amyl alcohol internal standard solution. The sample bottle was placed on a solid-phase microextraction device at 55 °C and preheated for 15 min. The SPME extracted fiber head was aged at 250 °C at the GC-MS inlet until there was no impurity peak, and extraction was performed for 30 min. Then, the samples were analyzed at 250 °C for 3 min.

GC-MS analysis was performed using an Agilent 7890 A gas chromatograph coupled to an Agilent 5975C mass-selective detector (Agilent Technologies, USA) and equipped with a DB-WAX capillary column (30 m × 250 μm, 0.25 μm film thickness). The oven temperature was initially maintained at 50 °C for 1 min, increased to 120 °C at 5 °C/min and held for 2 min, and then increased to 240 °C at 10 °C/min and held for 5 min. Helium was used as the carrier gas at a constant flow rate of 1.0 mL/min, and injections were performed in splitless mode. The inlet and transfer-line temperatures were 220 and 240 °C, respectively. Mass spectra were acquired in full-scan mode over an *m*/*z* range of 35–500 using electron ionization at 70 eV. The ion-source and quadrupole temperatures were 230 and 150 °C, respectively.

Raw chromatographic data were processed using Agilent ChemStation software (version G1701EA). Automatic peak integration was conducted using a slope sensitivity of 100, a peak width of 0.05 min, an area-rejection threshold of 500, and a height-rejection threshold of 100. All integrated peaks were manually reviewed and corrected where necessary. Co-eluting peaks were resolved by manual spectral deconvolution using the ChemStation spectral-extraction function. Compounds were tentatively assigned by comparing their mass spectra with the NIST 11 library, using a minimum spectral match score of 80%. Because retention indices and authentic standards were not used, all compound assignments were considered tentative.

Volatile compounds were semi-quantified using 4-methyl-2-amyl alcohol as the internal standard, assuming a response factor of 1. The semi-quantitative abundance was calculated as:

(4) $$C_{i}=\frac{A_{i}}{A_{\mathrm{IS}}}\times \frac{C_{\mathrm{IS}}V_{\mathrm{IS}}}{m}$$

where *A*_*i*_ and *A*_*IS*_ are the peak areas of the target compound and internal standard, respectively; *C*_*IS*_ and *V*_*IS*_ are the concentration and added volume of the internal-standard solution; and *m* is the sample mass. Because compound-specific calibration curves and response factors were not established, the results are reported as μg 4-methyl-2-amyl alcohol equivalents/g DW rather than as absolute concentrations.

A procedural blank containing ultrapure water was analyzed before the sample sequence to monitor system background and possible contamination. No interfering peaks were detected within the retention-time windows of the tentatively assigned compounds. Column-bleed signals, including siloxanes, were identified by library matching and excluded from subsequent analysis. Although pooled quality-control samples were not analyzed, the retention time and peak-area response of the internal standard remained consistent across the sample sequence, with a peak-area relative standard deviation below 15%. Three independently prepared biological replicates were analyzed for each drying condition, with one GC-MS injection per biological replicate. Statistical analyses were based on the three biological replicates, and the results are expressed as mean ± standard deviation.

#### 2.6.5 E-nose and E-tongue analysis

A PEN3 electronic nose (Airsense Analytics GmbH, Schwerin, Germany) was used following a previously published method (Liao et al., 2025). A 10 g sample was weighed into a 40 mL headspace bottle fitted with a Teflon cap. After 30 min at 37 °C, the headspace was injected into the device at a speed of 400 mL/min. The detection time was 80 s. The maximum output data for each sensor were used for further analysis.

The samples were analyzed using an electronic tongue (SA402B, Insent, Japan). The sample was diluted at a ratio of 1:10 with water, mixed using a food processor, centrifuged at 8000 ×g for 5 min, and the supernatant was filtered. The filtrate (70 mL) was then subjected to electronic tongue testing.

### 2.7 Statistical analysis

Origin 2022 (OriginLab, USA) and SPSS 22.0 (SPSS, Inc., Chicago, IL, USA) were used for data plotting and statistical analyses, respectively. Residual normality was assessed using the Shapiro-Wilk test and Q-Q plots, and homogeneity of variance was examined using Levene's test. The limited number of independent replicates *n* = 3 reduced the power of these diagnostic tests; nevertheless, the results indicated that the assumptions of normality and homogeneity of variance were generally satisfied for all reported parameters. Accordingly, one-way analysis of variance (ANOVA) followed by Duncan's multiple-range test was applied for group comparisons. A *p*-value of less than 0.05 was considered statistically significant, and exact *p*-values are reported where applicable. All experiments were performed in triplicate, and the data are presented as mean ± standard deviation (SD). For multivariate analyses (e.g., principal component analysis), peak areas of tentatively assigned compounds were imported into the free online Omicshare platform (https://cloud.metware.cn).

## 3 Results and discussion

### 3.1 Drying process optimization and drying characteristics

#### 3.1.1 Drying process optimization

The pre-temperature, post-temperature, and loading capacity were selected as the test factors, and a three-factor, three-level response surface test was conducted with browning degree *Y*_*1*_ and SEC *Y*_*2*_ as the response values. The experimental results of the responses were analyzed and the results obtained for browning degree and SEC under different drying conditions (pre-temperature A, post-temperature B and loading capacity C) using a Box-Behnken experimental design are presented in Table S1. The results were fitted to quadratic polynomial model equations and multiple regression coefficients were generated for all the responses using the method of least squares (MLS).

(5) $$Y_{1}=0.437+0.047875\;A+0.0065B+0.166125C-0.00375\mathrm{AB}+0.1015\;\mathrm{AC}+0.03225\;\mathrm{BC}+0.19525A^{2}+0.145B^{2}+0.29425C^{2}$$

(6) $$Y_{2}=4.217916+0.0745\;A+0.0274B-0.33090875C+0.713\mathrm{AB}+0.0090\;\mathrm{AC}-0.0329\;\mathrm{BC}+0.62179325A^{2}+0.26104075B^{2}+1.15363575C^{2}$$

The results of variance analysis of the single-index regression equations showed that the quadratic model was significant for all the analyzed indexes (*p* < 0.0001, Table S2). In the *Y*_*1*_ model, the determining coefficient *R*^*2*^ was 0.9873, and the model fitted well. According to the analysis of variance, we concluded that the influence of pre-temperature (A), post-temperature (B), and loading capacity(C) on the browning degree of figs was in the order B > A > C. In the *Y*_*2*_ model, the linear regression was extremely significant (*p* < 0.001), but the fitting term was not significant (*p* = 0.9771 > 0.01), with *R*^*2*^ = 0.9425. The regression model fitted the actual data well; therefore, this model can be used to predict *Y*_*2*_. According to the analysis of variance, the influence of pre-temperature (A), post-temperature (B) and loading capacity (C) on the SEC of figs was in the order C > A > B in decreasing order of influence.

After optimizing the extreme points of several response variables, the optimal parameters of the VTD process were determined as follows: A pre-temperature of 54.57 °C, a post-temperature of 79.84 °C, and a loading capacity of 2.73 g/dm^2^. Considering the feasibility and operability of actual production, these parameters were fine-tuned to a pre-temperature of 55 °C, a post-temperature of 80 °C, and a loading capacity of 2.73 g/dm^2^. Validation was then conducted. The adjusted browning degree and SEC were 0.44 and 4.17 kWh/kg (equivalent to 6.62 kWh/kg water removed), respectively. Comparing the actual production results with the model predicted values, we found that the relative errors were 0.91% and 0.96%, respectively, which were both far below the allowable range of 5% error, indicating that the optimized parameter combinations were feasible and accurate under the tested conditions. Compared with the traditional fig drying process (constant temperature of 60 °C), the optimized process conditions could reduce the browning degree and SEC by 22.8% and 20.4%, respectively, and thus could be used for the production of high-quality figs.

#### 3.1.2 Drying characteristics analysis

**Fig. 1:**
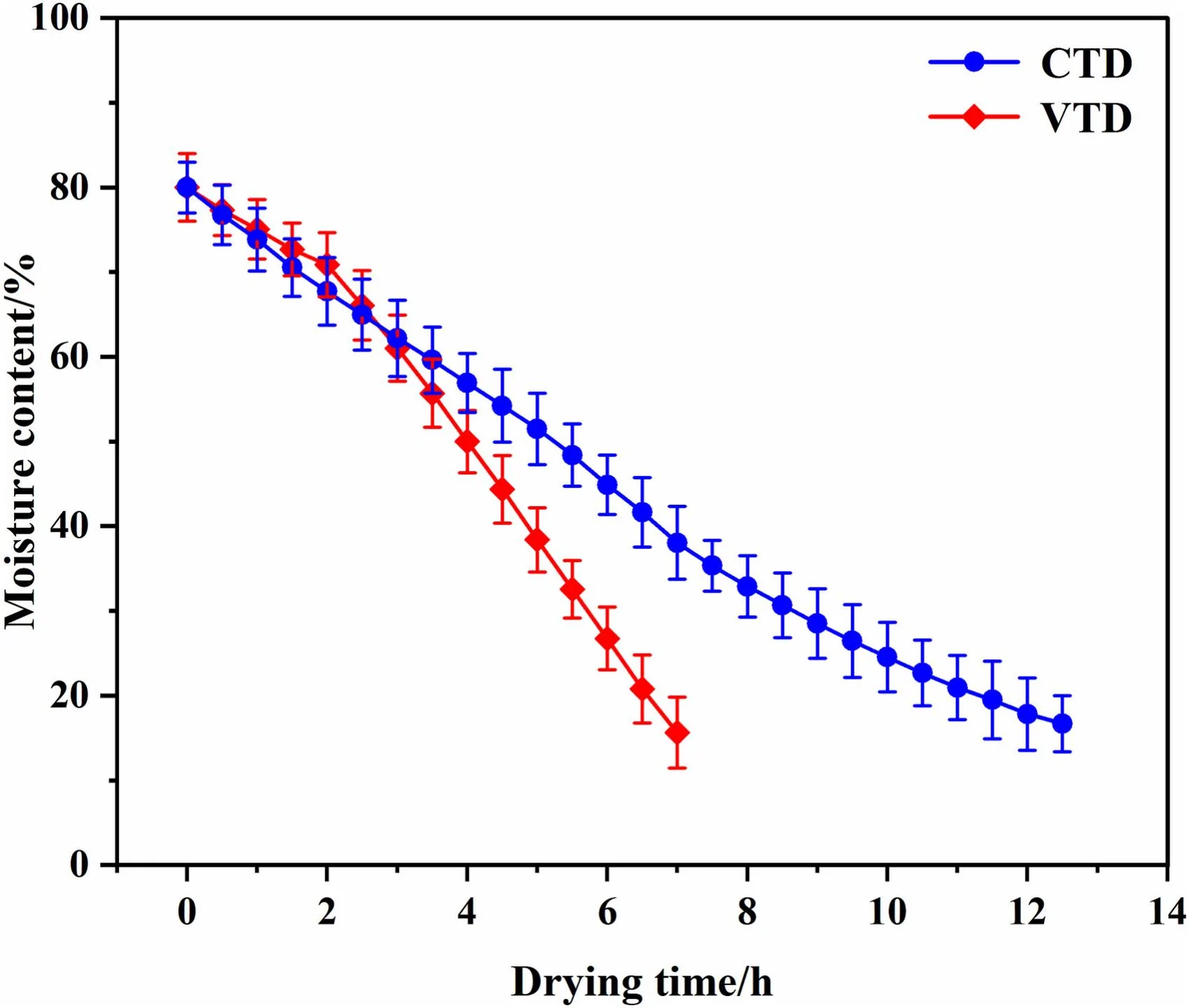
Drying characteristic curve of figs with different drying methods. CTD, constant temperature drying; VTD, variable temperature drying.

Drying temperature is the main factor affecting drying kinetics. According to our experimental results, figs can maintain a better color and luster when dried at 50 °C and 60 °C, and the higher the temperature, the lower SEC. Therefore, in this study, we chose a constant temperature of 60 °C as the control group, and carried out CTD and VTD tests on figs. The drying characteristic curve is shown in Fig. 1. Compared with CTD, VTD shortened the drying time by 44.8%, possibly because the staged temperature profile altered the moisture-removal conditions during drying. In the early stage of VTD, the low-temperature stage made the surface less prone to crusting, and the free water in the samples was continuously evaporated. In the late stage of VTD, the high-temperature air provided a higher external heat flux, which accelerated the diffusion of water inside the figs, thus increasing the drying rate. A previous study (Liao et al., 2024) explored the effects of different drying methods on the drying characteristics of jujube and found that the water vapor partial pressure in the air, as well as the water vapor partial pressure difference between the surface and the interior of the material, increased because of the gradient warming of VTD, which increased the rate of water evaporation and shortened the drying cycle. These results are consistent with our findings.

### 3.2 Effects on physical properties and microstructure

#### 3.2.1 Surface color

Color is a key indicator for evaluating the quality of dried products, and its variation is primarily attributed to the thermal degradation of polyphenols and chlorophyll, as well as enzymatic and non-enzymatic browning reactions (Maillard reaction) (Wang et al., 2025). As shown in Table S3, both CTD and VTD significantly affected the color parameters of figs (*p* < 0.05). With decreasing moisture content, the L* and b* values of figs under both drying methods decreased, whereas the a* value and *∆E* increased. Compared with CTD, VTD exhibited superior color retention, with reductions in L* and b* values decreased by 6.76% and 7.56%, respectively, indicating improved lightness and chromatic attributes. This effect can be attributed to the dynamic temperature control strategy of VTD, which shortens the drying duration and reduces thermal exposure, thereby limiting pigment degradation and phenolic oxidation. Similar findings were reported by Liu et al. (2021) in their study on the effects of drying temperature on the drying kinetics, color, and texture of jujube.

The total color difference was used to quantify overall color variation, with lower *∆E* values indicating better product quality (Liu et al., 2021). The *∆E* values of figs subjected to CTD and VTD were 22.61 and 17.95, respectively. Compared with CTD, the *∆E* of VTD figs decreased by 20.61%, which is associated with the shorter drying time. The shorter overall drying time in VTD may have reduced cumulative oxidation and pigment degradation, contributing to the better color retention. However, it should be noted that HMF, melanoidins, and other specific non-enzymatic browning markers were not measured in this study. Therefore, the color changes observed should not be attributed specifically to Maillard browning, as the measured color parameters (L, a, b*, ΔE) reflect the combined effects of multiple processes, including enzymatic oxidation, pigment degradation, and non-enzymatic reactions. In contrast, the prolonged drying time in CTD enhanced ascorbic acid oxidation, and thermal degradation of natural pigments, resulting in surface darkening and deterioration of the natural color (Hu et al., 2024). These results are consistent with those reported by Fu et al. (2026), who demonstrated that short-term high-temperature treatment effectively reduces browning in lanxangia tsao-ko. The dynamic temperature regulation in VTD effectively suppressed browning and was associated with improved instrumental color characteristics, which is in agreement with the response surface methodology results, further supporting the reliability of the optimized process under the conditions tested.

#### 3.2.2 Texture analysis

Texture properties are critical indicators for evaluating the textural characteristics and palatability of dried figs. A texture analyzer enables objective quantification by simulating the oral mastication process (Liu et al., 2021). As shown in Table S3, with the progression of drying and the reduction in moisture content, hardness, adhesiveness, gumminess, and chewiness exhibited an overall fluctuating increase, whereas springiness and cohesiveness increased continuously. Compared with fresh fruit, all these parameters were significantly elevated in dried figs, which is associated with surface hardening and internal structural densification induced by drying (Liao et al., 2024). Notably, at the end of drying, hardness, adhesiveness, gumminess, and chewiness reached their maximum values. Compared with CTD, VTD significantly reduced hardness (28.4%), adhesiveness (67.5%), gumminess (11.5%), and chewiness (10.4%) of dried figs, while no significant differences were observed in springiness and cohesiveness between the two methods (*p* > 0.05). The dynamic temperature control in VTD optimized heat and mass transfer rates and effectively shortened thermal exposure time, thereby mitigating tissue hardening caused by high-temperature oxidation and excessive dehydration, and maintaining a relatively porous structure in the dried product. Similar results have been reported for red beetroot (Milanovic et al., 2025), shiitake mushrooms (Zhang et al., 2022), and jujuba (Liao et al., 2024) under variable-temperature drying conditions.

#### 3.2.3 Microstructure

**Fig. 2:**
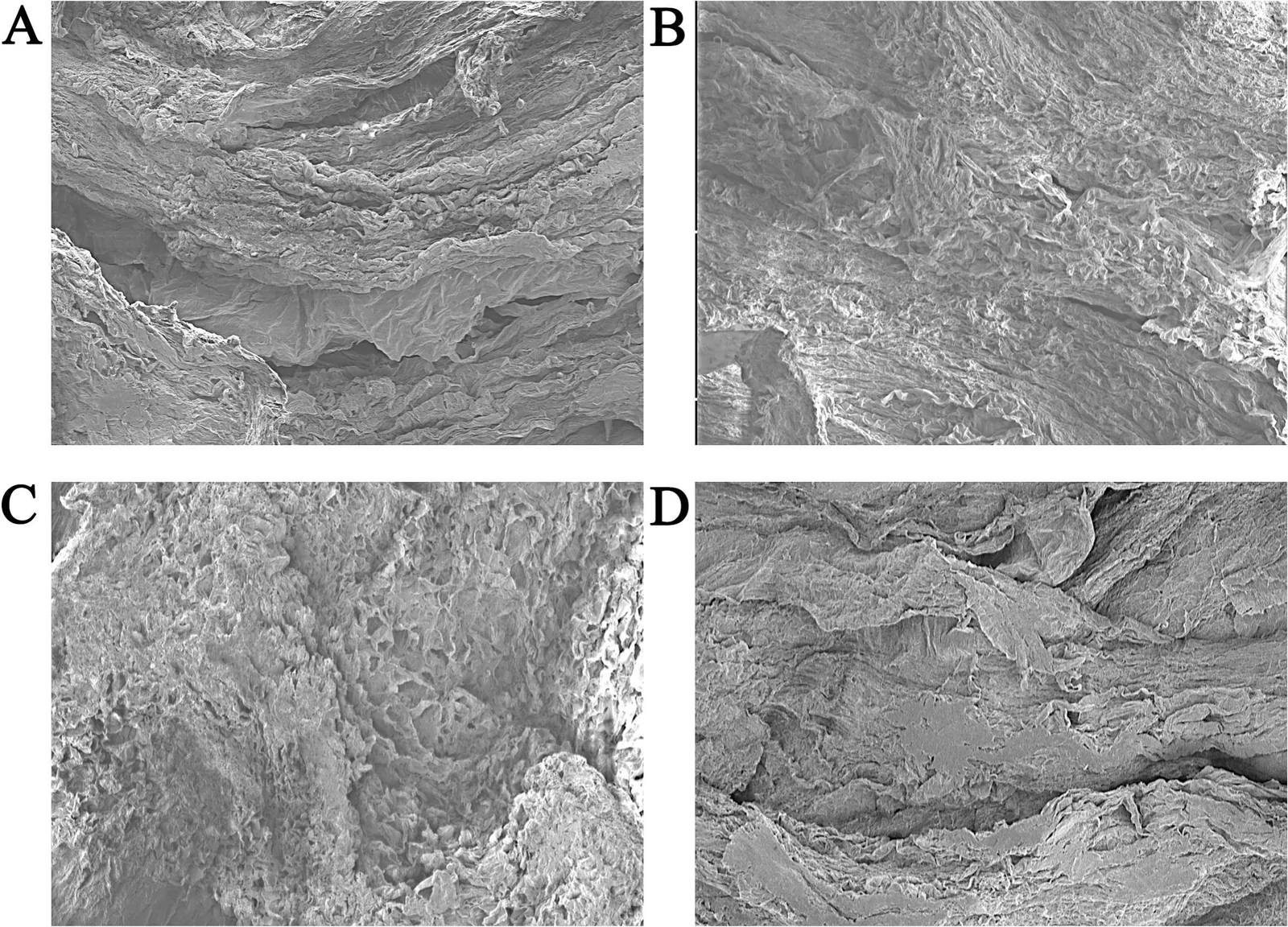
SEM images of figs with different drying methods. A, FS, fresh sample; B, VT-V, variable temperature drying-variable temperature point sample; C, VT-E, variable temperature drying-end point sample; D, CT-E, constant temperature drying-end point sample. SEM: scanning electron microscope.

Microstructure is a key intrinsic factor determining the texture and sensory quality of figs, and scanning electron microscopy (SEM) enables direct observation of cellular structural changes during drying. As shown in Fig. 2, fresh figs (Fig. 2A) exhibited well-organized and regularly arranged cells. The variable temperature drying-variable temperature point sample (VT-V) (Fig. 2B) showed only slight shrinkage compared with the fresh sample (FS), with no evident morphological differences. With continuous moisture loss, pronounced cell collapse and shrinkage were observed in the fully dried samples (variable temperature drying-end point sample (VT-E) and constant temperature drying-end point sample (CT-E); Fig. 2C and D), resulting in a more compact structure.

The CT-E sample exhibited a highly flattened morphology, which is associated with structural compaction caused by tissue collapse and shrinkage during drying. Moreover, the prolonged drying time in CTD promoted contraction and aggregation of polysaccharides and proteins, leading to a denser structure and more severe tissue shrinkage. This observation is consistent with the significantly higher hardness of the CT-E sample reported in Section 3.2.2. In contrast, the VT-E sample presented a looser and smoother structure, with less cellular deformation and better preservation of intercellular spaces. This can be attributed to the dynamic temperature regulation in VTD, which markedly shortened the overall drying time and reduced thermal accumulation, thereby alleviating excessive collapse and shrinkage (Yao et al., 2022). These findings are consistent with the results of color and texture analyses, further suggesting the advantage of VTD in preserving microstructure and improving overall quality. Similar observations have been reported for paddy rice (Xu et al., 2022) and jujube (Liao et al., 2024) subjected to VTD, which exhibited better microstructural integrity and spatial organization.

### 3.3 Retention of nutritional compounds and antioxidant capacity

**Fig. 3:**
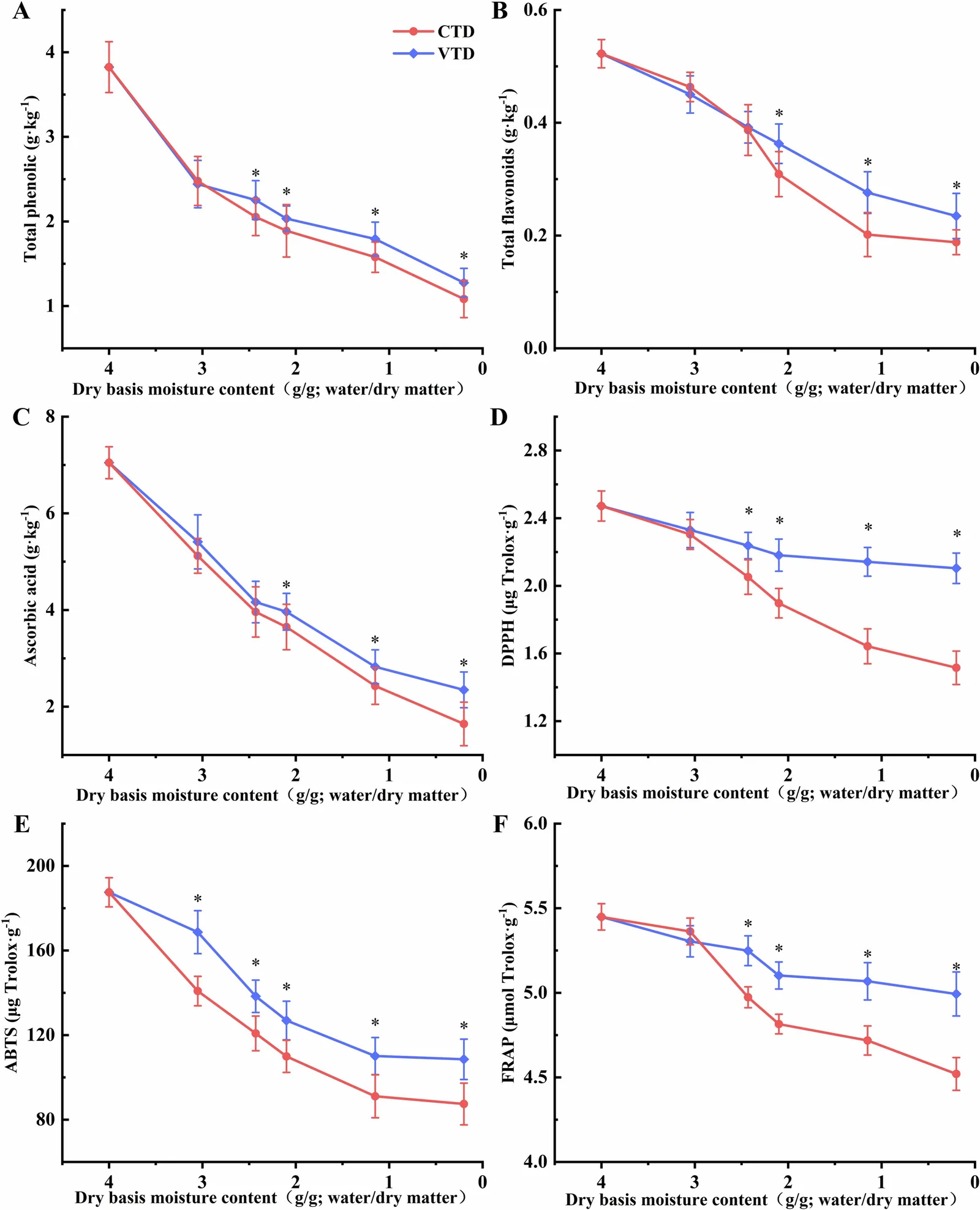
Effects of different drying methods on antioxidant substances and antioxidant capacity of figs. A, total phenolic; B, total flavonoids; C, ascorbic acid; D, DPPH free radical scavenging ability; E, ABTS free radical scavenging ability; F, FRAP total antioxidant capacity. CTD, constant temperature drying; VTD, variable temperature drying. * indicates significant difference at *p* < 0.05, *n* = 3 for each treatment.

The contents of antioxidant compounds and the antioxidant capacity of the samples are presented in Fig. 3. During the drying process, both the levels of antioxidant compounds and the antioxidant capacity of figs decreased significantly. The overall antioxidant capacity was in the order: FS > VTD > CTD. The contents of total phenolics, total flavonoids, and ascorbic acid in VTD-treated figs were significantly higher than those in CTD-treated samples by 0.19 g/kg, 0.05 g/kg, and 0.71 g/kg.

Phenolic compounds are important secondary metabolites in fruits, exhibiting antioxidant, anti-inflammatory, anticancer, and metabolic regulatory functions (Jafari et al., 2025). As shown in Fig. 3A, drying led to the degradation of polyphenols in figs, and their content gradually decreased with the reduction in moisture content. Compared with FS, total phenolic content decreased by 2.55 and 2.74 g/kg in VTD- and CTD-treated figs, respectively, which is associated with the susceptibility of phenolic compounds to degradation and oxidation under aerobic and thermal conditions. At the end of drying, the retention of total phenolics in VTD-treated figs was 17.59% higher than that in CTD-treated samples, which may be related to the higher drying efficiency of VTD. Previous studies have shown that phenolic compounds in cranberry (Zielinska & Zielinska, 2019) and jujube (Liu et al., 2022) are more affected by drying time (i.e., exposure to oxygen) than by temperature, and that high-temperature, short-time VTD processes reduce oxidative degradation of phenolics, consistent with the present findings.

Flavonoids also exhibit antioxidant activity by donating hydrogen atoms to neutralize reactive oxygen species (Amarowicz et al., 2004). As shown in Fig. 3B, the total flavonoid content of figs decreased significantly during drying (*p* < 0.05). Compared with FS, total flavonoid content decreased by 0.29 g/kg and 0.33 g/kg in VTD- and CTD-treated samples, respectively. This reduction is mainly associated with the disruption of cellular barriers during dehydration, which facilitates the leakage of intracellular bioactive compounds and their subsequent irreversible oxidation and thermal degradation (Liao et al., 2025). Compared with CTD, VTD improved the retention of total flavonoids by 27.78%, which may be attributed to the shorter drying duration and reduced degradation rate. Similar findings have been reported by Hu et al. (2023), who observed that hot-air drying resulted in greater flavonoid loss in coffee peel compared with vacuum freeze-drying, and that flavonoid degradation was associated with prolonged drying time and excessive temperature.

Ascorbic acid is one of the most important antioxidant compounds in fruits and is prone to oxidation to dehydroascorbic acid during drying, followed by decarboxylation and cyclization reactions, ultimately forming 3-hydroxy-2-pyrone and 2-furoic acid (Ordóñez-Santos et al., 2017). In this study, compared with FS, ascorbic acid content decreased by 4.70 g/kg and 5.41 g/kg in VTD- and CTD-treated figs, respectively, with a 43.29% higher retention in VTD samples (Fig. 3C). Ascorbic acid is sensitive to temperature, and excessive heat can alter its structure, while prolonged processing time promotes its oxidation (Zhao, Al-Dalali, et al., 2025; Zhao, Bian, et al., 2025; Zhao, Liu, et al., 2025). The loss of ascorbic acid in figs observed in this study may be attributed to high drying temperatures, extended drying duration, and increased exposure to oxygen due to airflow, leading to irreversible degradation. Compared with CTD, VTD resulted in higher ascorbic acid retention at the drying endpoint.

Due to the complexity of plant matrices, a single analytical method is insufficient to comprehensively evaluate antioxidant capacity (Valadez-Carmona et al., 2016). Therefore, three assays - DPPH radical scavenging activity, ABTS radical scavenging activity, and ferric reducing antioxidant power (FRAP)-were employed in this study. As shown in Fig. 3D-3F, all antioxidant capacity indices decreased after drying. Compared with FS, DPPH values decreased by 100.12 and 78.99 μg Trolox/g, ABTS values decreased by 0.95 and 0.37 μg Trolox/g, and FRAP values decreased by 0.93 and 0.46 μmol Trolox/g in CTD- and VTD-treated figs, respectively. Although antioxidant capacity declined under both drying methods, VTD better preserved antioxidant activity than CTD. In addition, it is also possible that newly formed reducing compounds, including some Maillard-derived products, contributed to the DPPH, ABTS, or FRAP responses. Previous studies have reported that thermal drying reduces antioxidant capacity in cabbage (Xu et al., 2020) and kiwifruit (Ao et al., 2025), primarily due to the degradation of antioxidant compounds such as phenolics, flavonoids, and ascorbic acid under high thermal loads, which is consistent with the present results. Overall, thermal processing exerts negative effects on bioactive compounds in plant materials, and prolonged drying leads to substantial losses in antioxidant capacity and active constituents. In this study, VTD showed superior performance in preserving bioactive compounds in figs.

### 3.4 Evolution of non-volatile profiles

#### 3.4.1 Soluble sugars

Soluble sugars are not only important nutritional components of figs but also key precursors for thermally induced flavor-forming reactions such as the Maillard reaction (Zhao, Al-Dalali, et al., 2025; Zhao, Bian, et al., 2025; Zhao, Liu, et al., 2025). As shown in Table 1, the total soluble sugar content in fresh samples was 782.17 g/kg DW, with glucose and fructose as the predominant components, accounting for 53.1% and 45.2%, respectively, while sucrose (10.28 g/kg DW) and galactose (3.00 g/kg DW) were present at relatively low levels. These results are consistent with those reported by Slatnar et al. (2011). No significant difference in total sugar content was observed between the VT-V sample and the fresh sample (*p* > 0.05); however, sucrose content decreased by 70.6%. This reduction may be attributed to the activation of relevant enzymes during the initial stage of drying, induced by increasing temperature, which promotes the conversion of sucrose into glucose and fructose (Liao et al., 2024). Consequently, slightly higher levels of glucose and fructose were observed in VT-V compared with the fresh sample.

**Table 1:** Effects of different drying methods on non-volatile flavor compounds of figs.

Indicators	FS	VT-V	VT-E	CT-E
Soluble sugars (g/kg DW)				
Galactose	3.00 ± 0.61^a^	2.62 ± 0.86^ab^	2.12 ± 0.78^b^	1.96 ± 0.77^b^
Glucose	415.59 ± 38.53^a^	424.98 ± 37.37^a^	365.86 ± 40.42^b^	372.85 ± 33.06^b^
Fructose	353.32 ± 37.21^a^	357.60 ± 40.55^a^	362.71 ± 30.17^a^	345.15 ± 33.25^ab^
Sucrose	10.28 ± 2.41^a^	3.02 ± 0.19^d^	3.47 ± 0.94^c^	5.52 ± 0.04^b^
Total	782.17 ± 80.09^a^	788.22 ± 82.23^a^	734.16 ± 62.36^ab^	725.48 ± 80.34^b^

Free amino acids (μg/g DW)				
Histidine	133.26 ± 12.35^b^	147.82 ± 13.99^a^	120.54 ± 13.62^cd^	110.81 ± 10.53^d^
4-Hydroxy-L-proline	4.72 ± 0.33^c^	5.87 ± 0.45^b^	5.94 ± 0.39^b^	6.78 ± 0.56^a^
Arginine	224.87 ± 13.91^d^	305.46 ± 28.34^b^	260.18 ± 25.19^c^	325.86 ± 31.87^a^
Asparagine	7946.06 ± 659.90^a^	6633.10 ± 582.73^b^	4612.69 ± 476.36^d^	5165.98 ± 499.24^c^
Glutamine	4330.71 ± 397.75^a^	4147.90 ± 405.73^a^	1889.74 ± 184.61^c^	2480.51 ± 237.60^b^
Serine	1450.70 ± 137.13^a^	867.26 ± 79.55^bc^	727.01 ± 68.51^d^	803.44 ± 77.55^c^
Glycine	172.41 ± 16.21^b^	170.88 ± 13.86^b^	170.96 ± 16.66^b^	194.86 ± 18.78^a^
Aspartic acid	437.20 ± 45.14^a^	283.06 ± 25.18^d^	377.93 ± 35.17^b^	330.22 ± 32.89^c^
L-Citrulline	100.64 ± 11.92^a^	65.43 ± 7.77^b^	45.28 ± 5.57^cd^	47.06 ± 5.84^c^
Glutamic acid	102.31 ± 9.84^a^	33.32 ± 4.48^bc^	26.72 ± 3.95^d^	35.07 ± 4.15^b^
Threonine	259.67 ± 26.14^b^	294.30 ± 30.81^a^	236.75 ± 24.65^c^	286.88 ± 30.13^a^
Alanine	1835.42 ± 175.69^a^	1384.14 ± 144.24^b^	1215.29 ± 127.22^c^	1189.01 ± 106.77^d^
γ-Aminobutyric acid	861.63 ± 79.63^bc^	935.46 ± 88.95^b^	814.51 ± 83.38^c^	1093.73 ± 98.27^a^
Proline	2933.36 ± 276.61^a^	2927.97 ± 286.83^a^	2373.46 ± 191.14^c^	2719.66 ± 257.94^b^
L-Ornithine	33.71 ± 4.11^a^	20.31 ± 3.24^b^	14.69 ± 2.14^c^	14.14 ± 4.60^c^
D-2-Aminobutyric acid	6.39 ± 1.03^a^	5.43 ± 0.76^b^	2.89 ± 0.33^d^	3.80 ± 0.35^c^
Lysine	55.89 ± 6.12^c^	79.76 ± 8.28^b^	77.46 ± 6.54^b^	103.00 ± 9.74^a^
Cystine	2.66 ± 0.81^a^	0.56 ± 0.08^b^	0.05 ± 0.02^d^	0.27 ± 0.01^c^
Tyrosine	129.29 ± 11.39^c^	180.84 ± 17.20^ab^	172.57 ± 16.37^b^	189.03 ± 19.25^a^
Methionine	34.07 ± 3.92^a^	32.91 ± 3.45^ab^	34.29 ± 4.25^a^	33.12 ± 2.96^a^
Valine	205.41 ± 19.27^c^	265.52 ± 24.44^ab^	231.88 ± 28.49^b^	284.41 ± 30.16^a^
Isoleucine	141.91 ± 13.43^d^	193.79 ± 17.17^b^	171.68 ± 19.53^c^	208.80 ± 21.18^a^
Leucine	205.05 ± 21.73^c^	298.95 ± 30.87^bc^	329.99 ± 33.36^b^	366.97 ± 35.91^a^
Phenylalanine	144.45 ± 15.93^d^	203.76 ± 19.86^b^	196.23 ± 20.75^bc^	240.07 ± 23.84^a^
Tryptophan	49.73 ± 5.27^bc^	50.49 ± 4.92^b^	43.50 ± 4.72^c^	53.08 ± 6.11^a^
Total	21,801.51 ± 1957.42^a^	19,534.27 ± 1793.79^b^	14,152.22 ± 1325.63^d^	16,286.56 ± 1592.66^c^

Organic acids (μg/g DW)				
Mangiferonic acid	139.80 ± 12.61^bc^	130.91 ± 12.86^d^	177.24 ± 18.78^a^	145.87 ± 13.77^b^
Quinic acid	2439.25 ± 239.53^a^	2088.45 ± 214.37^b^	1498.27 ± 122.42^c^	1407.87 ± 133.06^c^
Malic acid	4778.60 ± 481.84^a^	3612.26 ± 352.43^b^	2086.88 ± 121.78^c^	1850.57 ± 149.25^d^
Succinic acid	187.51 ± 20.21^c^	231.19 ± 19.55^a^	213.05 ± 30.19^bc^	221.37 ± 23.25^b^
Oxalic acid	288.12 ± 30.41^b^	350.86 ± 36.19^a^	180.47 ± 22.94^c^	170.16 ± 20.04^d^
Citric acid	4835.52 ± 367.79^a^	4771.42 ± 397.18^ab^	2512.49 ± 309.94^b^	2578.69 ± 309.44^b^
Total	12,668.81 ± 1375.09^a^	11,185.09 ± 1274.23^b^	6668.40 ± 629.36^c^	6374.52 ± 827.34^c^

**Note:** Values are mean ± SD. *n* = 3 for each treatment. The different representations of small letters in the same index are significantly different (*p* < 0.05). FS, fresh sample; VT-V, variable temperature drying-variable temperature point sample; VT-E, variable temperature drying-end point sample; CT-E, constant temperature drying-end point sample.

The total sugar contents of VT-E and CT-E figs were 734.16 g/kg DW and 725.48 g/kg DW, respectively. Compared with VT-V figs. (424.98 g/kg DW), the glucose content in VT-E figs. (365.86 g/kg DW) decreased significantly (*p* = 0.036). In contrast, no significant difference was observed in fructose content between VT-E (362.71 g/kg DW) and VT-V (357.60 g/kg DW) samples (*p* > 0.05), indicating that fructose remained relatively stable during the later stage of drying. Glucose decreased significantly between VT-V and VT-E, whereas fructose remained relatively stable. This pattern is consistent with the possible involvement of glucose in thermally induced reactions, including Maillard-type and sugar-degradation reactions. However, reducing-sugar kinetics and pathway-specific products were not measured; therefore, preferential consumption of glucose through a specific flavor-forming pathway cannot be concluded. This observation is consistent with the findings of Liao et al. (2024), who reported similar effects of variable-temperature drying on sugar composition and related enzymatic activity in jujube.

#### 3.4.2 Free amino acids

Free amino acids in plant-based foods play a dual role in the diet, providing essential amino acids to the human body while also participating in certain reactions during food processing, such as the Maillard reaction (Fu et al., 2026). Table 1 shows the changes in the content of free amino acids in figs during different drying processes. A total of 25 free amino acids were detected in fresh figs, with a total content of 21,801.51 μg/g DW. The three amino acids with the highest content were asparagine, glutamine, and proline, with contents of 7946.06, 4330.71, and 2933.36 μg/g DW, respectively. As the moisture content decreased, the total free amino acid content gradually decreased. The CT-E fig samples contained a higher total amount of free amino acids (16,286.56 μg/g DW) than the VT-E samples (14,152.22 μg/g DW). This may be due to the higher temperature during the later stages of VTD, where free amino acids acted as precursors in the Maillard reaction, leading to a reduction in the total content of free amino acids. This finding is consistent with the results of Liao et al. (2025) and Liu et al. (2022), which showed that as drying temperature increased, the free amino acid content in jujube gradually decreased.

#### 3.4.3 Organic acids

Fruits typically contain abundant organic acids, such as citric acid, malic acid, and oxalic acid, which are not only key flavor components but also play important nutritional roles (Slatnar et al., 2011). Table 1 presents the six organic acids (mangiferonic acid, quinic acid, malic acid, succinic acid, oxalic acid, and citric acid) with the highest content detected in figs, along with their respective concentrations. The organic acid content in FS reached 12,668.8 μg/g DW, with citric acid (4835.52 μg/g DW) being the most abundant, followed by malic acid (4778.60 μg/g DW) and quinic acid (2439.25 μg/g DW). As the moisture content decreased, the total organic acid content gradually declined, which is attributed to their oxidation and decarboxylation during the hot air drying process (Liu et al., 2022). The organic acid content in the VT-E sample (6668.40 μg/g DW) was higher than in the CT-E sample (6374.52 μg/g DW), which may be related to the shorter high-temperature exposure during VTD, effectively reducing the cumulative thermal degradation of organic acids. It may also be due to the disruption of cellular compartmentalization by high temperatures in the later stages, which released some bound organic acids (Liao et al., 2024). In contrast, although the CT-E sample had a slightly lower temperature, prolonged heat accumulation led to more severe decarboxylation and degradation of major organic acids such as malic acid and citric acid.

### 3.5 Evolution of volatile profiles

**Fig. 4:**
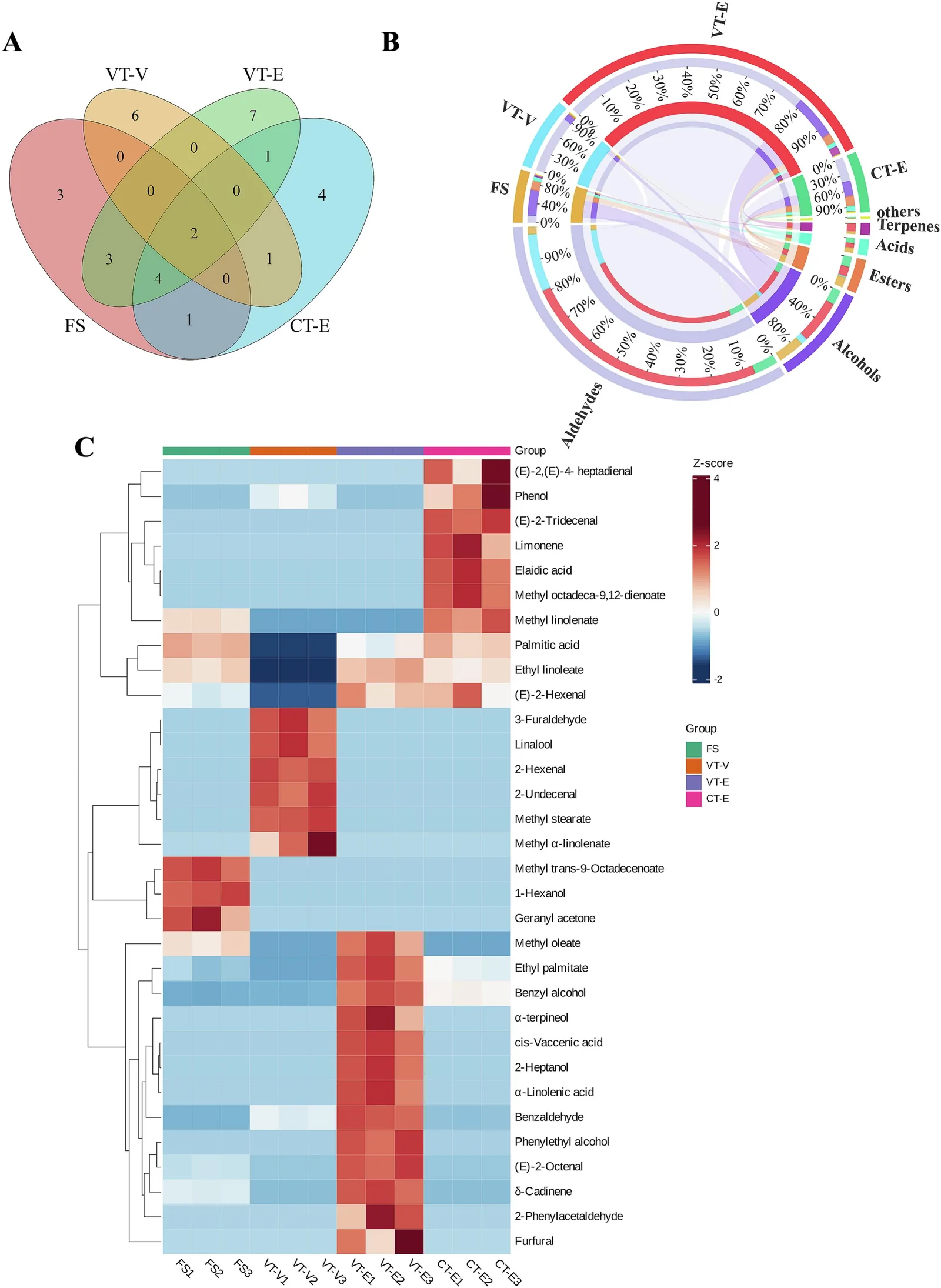
The volatile compounds in figs at different drying methods based on GC-MS data. A, Venn diagram; B, Circos plot; C, Heat map. FS, fresh sample; VT-V, variable temperature drying-variable temperature point sample; VT-E, variable temperature drying-end point sample; CT-E, constant temperature drying-end point sample.

Volatile compounds in the FS, VT-V, VT-E, and CT-E samples were analyzed by HS-SPME-GC-MS (Table 2). Using a spectral match score above 80% as the screening criterion, 32 compounds were tentatively assigned, comprising 10 aldehydes, 4 acids, 6 alcohols, 8 esters, 3 terpenes, and 1 other compound. Because the compounds were semi-quantified with a single internal standard and compound-specific response factors were not determined, the values represent internal-standard-based semi-quantitative abundances rather than absolute concentrations. The summed abundances were 6.08, 8.31, 39.78, and 7.01 μg/g DW in FS, VT-V, VT-E, and CT-E, respectively. VT-E had the highest number of unique volatile compounds (7), followed by VT-V (6), CT-E (4), and FS (3) (Fig. 4A). VT-E had a significantly higher summed abundance than the other samples (*p* < 0.05), whereas FS, VT-V, and CT-E did not differ significantly from one another (*p* > 0.05). This difference was driven primarily by aldehydes, especially benzaldehyde, and should not be interpreted as a uniform increase in all volatile compounds or as direct evidence of improved sensory quality.

**Table 2:** Tentatively assigned volatile compounds and their internal-standard-based semi-quantitative abundances in fresh and dried figs.

	Compounds	Cas	Content (μg/g DW)				Odor description
			FS	VT-V	VT-E	CT-E	
	Aldehydes						
1	(E)-2,(E)-4- heptadienal	4313-03-5	–	–	–	0.10 ± 0.06	Green, Fatty, Cucumber
2	2-Hexenal	505–57-7	–	0.15 ± 0.01	–	–	Green, Leafy, Apple
3	(E)-2-Hexenal	6728-26-3	0.66 ± 0.07^b^	–	1.19 ± 0.24^a^	1.22 ± 0.53^a^	Green, Leafy, Apple
4	(E)-2-Octenal	2548-87-0	0.06 ± 0.01^b^	–	0.61 ± 0.05^a^	–	Green, Fatty, Nutty
5	(E)-2-Tridecenal	7069-41-2	–	–	–	0.21 ± 0.04^c^	Waxy, Citrus, Floral
6	2-Undecenal	2463-77-6	–	0.08 ± 0.01	–	–	Waxy, Fatty, Floral
7	3-Furaldehyde	498–60-2	–	0.07 ± 0.01	–	–	Sweet, Bready, Caramel
8	Benzaldehyde	100–52-7	0.16 ± 0.02^d^	6.98 ± 0.74^b^	27.05 ± 1.49^a^	1.48 ± 0.12^c^	Almond, Cherry, Sweet
9	2-Phenylacetaldehyde	122–78-1	–	–	1.34 ± 0.61	–	Floral, Honey, Sweet
10	Furfural	98–01-1	–	–	1.28 ± 0.86	–	Sweet, Caramel, Bread
	Acids						
11	α-Linolenic acid	463–40-1	–	–	0.06 ± 0.01	–	Faint, Fatty
12	Elaidic acid	112–79-8	–	–	–	0.19 ± 0.03	Faint, Fatty
13	cis-Vaccenic acid	506–17-2	–	–	0.18 ± 0.02	–	Faint, Fatty
14	Palmitic acid	57–10-3	0.52 ± 0.02^a^	–	0.33 ± 0.04^b^	0.47 ± 0.06^a^	Faint, Waxy
	Alcohols						
15	Linalool	78–70-6	–	0.22 ± 0.03	–	–	Floral, Lavender, Citrus
16	1-Hexanol	111–27-3	2.71 ± 0.18	–	–	–	Green, Grassy, Fatty
17	2-Heptanol	14,049–11-7	–	–	1.09 ± 0.14	–	Floral, Woody
18	α-terpineol	1517-69-7	–	–	0.03 ± 0.01	–	Floral, Sweet, Rose
19	Benzyl alcohol	100–51-6	0.56 ± 0.04^c^	0.62 ± 0.02^c^	3.95 ± 0.28^a^	1.88 ± 0.07^b^	Floral, Sweet, Fruity
20	Phenylethyl alcohol	60–12-8	–	–	0.42 ± 0.05	–	Floral, Rose, Honey
	Esters						
21	Methyl linolenate	301–00-8	0.29 ± 0.02^b^	–	–	0.51 ± 0.07^a^	Faint, Fatty
22	Methyl octadeca-9,12-dienoate	2462-85-3	–	–	–	0.16 ± 0.04^c^	Faint, Fatty
23	Methyl oleate	2566-97-4	0.14 ± 0.02^b^	–	0.25 ± 0.05^a^	–	Faint, Fatty
24	Methyl α-linolenate	17,309–05-6	–	0.02 ± 0.01	–	–	Faint, Fatty
25	Methyl trans-9-Octadecenoate	1937-62-8	0.09 ± 0.01	–	–	–	Faint, Fatty
26	Ethyl palmitate	628–97-7	0.03 ± 0.01^c^	–	0.25 ± 0.03^a^	0.08 ± 0.01^b^	Waxy, Floral
27	Methyl stearate	112–39-0	–	0.14 ± 0.01	–	–	Waxy, Floral
28	Ethyl linoleate	5129-60-2	0.62 ± 0.05^a^	–	0.73 ± 0.05^a^	0.56 ± 0.03^a^	Waxy, Fatty
	Terpenes						
29	Geranyl acetone	17,699–14-8	0.03 ± 0.01	–	–	–	Woody, Pine
30	Limonene	138–86-3	–	–	–	0.03 ± 0.01	Citrus, Lemon, Orange
31	δ-Cadinene	483–76-1	0.21 ± 0.01^b^	–	1.02 ± 0.09^a^	–	Woody, Spicy
	Others						
32	Phenol	108–95-2	–	0.03 ± 0.01^b^	–	0.12 ± 0.05^a^	Phenolic, Medicinal, Sweet
	Total		6.08 ± 0.19^b^	8.31 ± 0.04^b^	39.78 ± 1.88^a^	7.01 ± 0.58^b^	

**Note:** FS, fresh sample; VT-V, variable temperature drying-variable temperature point sample; VT-E, variable temperature drying-end point sample; CT-E, constant temperature drying-end point sample.Values are expressed as μg 4-methyl-2-amyl alcohol equivalents/g DW. These values are semi-quantitative and were calculated assuming a response factor of 1. Different superscript lowercase letters in the same row indicate significant differences at *p* < 0.05. Values are mean ± SD. *n* = 3 for each treatment.“–” indicates that the compound was not detected under the analytical conditions.

Aldehydes showed the clearest treatment-dependent differences. Benzaldehyde was detected in all four samples and increased from 0.16 μg/g DW in FS to 6.98 μg/g DW at VT-V and 27.05 μg/g DW at VT-E, compared with 1.48 μg/g DW in CT-E. It accounted for approximately 68% of the summed abundance in VT-E and therefore strongly influenced the total. The VT-E sample also contained 2-phenylacetaldehyde (1.34 μg/g DW) and furfural (1.28 μg/g DW), which were not detected in FS or CT-E. Benzaldehyde and 2-phenylacetaldehyde are commonly associated with amino-acid- and sugar-related thermal reactions, whereas furfural can arise from sugar degradation (Huang et al., 2023; Mathatheeranan et al., 2024; Zhao, Al-Dalali, et al., 2025; Zhao, Bian, et al., 2025; Zhao, Liu, et al., 2025). In contrast, the green-note compound (E)-2-hexenal was present at comparable abundances in VT-E (1.19 μg/g DW) and CT-E (1.22 μg/g DW), indicating that its abundance was not specifically increased by VTD.

The alcohol and ester profiles also differed among treatments. The fresh sample was characterized by 1-hexanol (2.71 μg/g DW), together with benzyl alcohol (0.56 μg/g DW), compounds associated with green, grassy, floral, and fruity odor descriptions. During drying, 1-hexanol was no longer detected, whereas benzyl alcohol increased to 0.62, 3.95, and 1.88 μg/g DW in VT-V, VT-E, and CT-E, respectively. The VT-E sample additionally contained 2-heptanol (1.09 μg/g DW), phenylethyl alcohol (0.42 μg/g DW), and alpha-terpineol (0.03 μg/g DW). Among the esters, ethyl linoleate was detected in FS, VT-E, and CT-E at 0.62, 0.73, and 0.56 μg/g DW, respectively, with no significant difference among these samples. Ethyl palmitate was higher in VT-E (0.25 μg/g DW) than in FS and CT-E, whereas several methyl esters occurred only in individual treatments. These patterns indicate treatment-dependent changes in the instrumental volatile profile, but their sensory importance cannot be established without compound-specific quantification, odor-activity analysis, and sensory evaluation.

The chord diagram (Fig. 4B) illustrates the relative associations between the four drying stages and the major classes of tentatively assigned volatile compounds. VT-E showed the strongest connections with aldehydes and alcohols, mainly because of the high abundance of benzaldehyde (27.05 μg/g DW), which accounted for approximately 68% of the total semi-quantitative abundance in this sample. 2-Phenylacetaldehyde and furfural were also detected only in VT-E, further distinguishing its aldehyde profile. The VT-E sample also showed associations with selected alcohols, esters, and terpenes, including benzyl alcohol, 2-heptanol, ethyl palmitate, ethyl linoleate, and δ-cadinene. In contrast, FS was characterized by 1-hexanol and several lipid-related esters, reflecting the fresh-fruit volatile profile. The VT-V profile was relatively weak and dispersed, indicating simultaneous loss of some fresh-tissue volatiles and appearance of several low-abundance compounds during intermediate drying. CT-E showed weaker overall connections than VT-E and was mainly associated with benzyl alcohol, benzaldehyde, (E)-2-hexenal, and ethyl linoleate. Notably, (E)-2-hexenal showed similar abundances in VT-E and CT-E, indicating that it was not specifically enhanced by VTD. Overall, the diagram represents compositional associations based on semi-quantitative abundances, rather than direct chemical pathways or sensory importance.

The occurrence and higher semi-quantitative abundances of benzaldehyde, 2-phenylacetaldehyde, and furfural in VT-E are consistent with compositional changes that may accompany Maillard-type, Strecker-related, and sugar-degradation reactions during the final 80 °C stage. However, the chord diagram shows treatment-associated compositional distributions rather than direct chemical conversion pathways. Therefore, these possible reaction routes remain inferential and require targeted experimental verification. The higher abundance of selected thermally associated volatiles in VT-E does not necessarily contradict its lower browning degree. Browning reflects the combined effects of enzymatic oxidation, pigment degradation, ascorbic acid oxidation, and the formation of high-molecular-weight colored products, whereas volatile aldehydes and furans may be formed or released during earlier or parallel stages of thermal reactions. A relatively short final exposure to 80 °C may therefore alter the abundance of selected volatile compounds without producing the cumulative color deterioration associated with prolonged drying. Nevertheless, because HMF, melanoidins, PPO, and POD were not measured, the relative contributions of enzymatic and non-enzymatic browning could not be resolved in the present study.

The heatmap (Fig. 4C) reveals distinct clustering patterns among treatments. FS and VT-V clustered closely, suggesting that the volatile profile remained relatively similar during the early drying stage. In contrast, VT-E was clearly separated from both FS and VT-V, indicating a marked reconstruction of the volatile profile during the final VTD stage. In VT-E, aromatic aldehydes and furans—including benzaldehyde, furfural, 3-furaldehyde, and 2-phenylacetaldehyde—were significantly enriched, corresponding to the high-intensity signal region in the heatmap, which is characteristic of thermally processed products. In contrast, the CT-E sample exhibited a relatively weak overall signal intensity and lacked any distinct compound aggregation region, suggesting limited volatile formation under constant-temperature drying.

Overall, VTD produced a volatile profile that differed from that of CTD at 60 °C under the tested conditions. However, these results should be interpreted as comparative and exploratory, given the semi-quantitative nature of the analysis and the tentative compound identification.

### 3.6 Electronic nose analysis

**Fig. 5:**
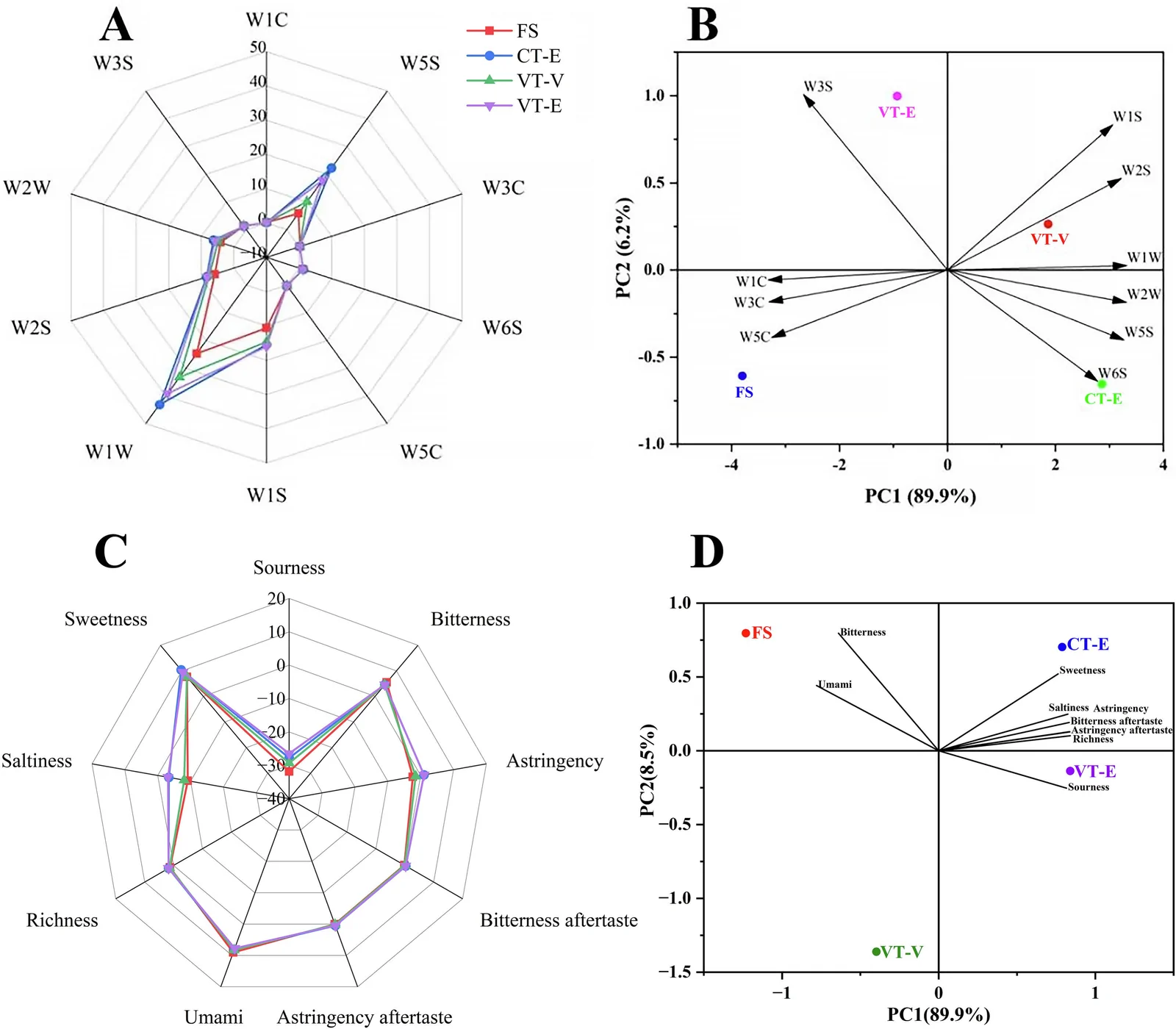
Multi-sensor evaluation of figs under different drying methods. A, Electronic nose radar plot; B, PCA from electronic nose; C, Electronic tongue radar plot; D, PCA from electronic tongue. FS, fresh sample; VT-V, variable temperature drying-variable temperature point sample; VT-E, variable temperature drying-end point sample; CT-E, constant temperature drying-end point sample.

Electronic nose is a quality control method that comprehensively evaluates the total odor information of the examined samples (Liao et al., 2025). The signal trends of different fig samples were similar (Fig. 5A), all of which showed higher responses to the W1W, W5S, W2S and W2W sensors, suggesting that sulfur compounds, nitrogen oxides and aromatic compounds contributed more to the odor of the fig samples. Significant differences (*p* < 0.05) were found among the FS, VT-V, VT-E and CT-E, and these differences might be related to the production of different volatiles under different drying conditions and times.

Principal component analysis (PCA) is an advanced multivariate statistical analysis technique. This technique is able to identify and retain key information from the many complex and imperceptible variables in the original samples, and to succinctly and accurately reveal the underlying patterns and variability among the samples by transforming them into a small number of principal component factors (Li et al., 2019). Herein, PCA of the dataset of electronic nose response values of fig samples dried using different drying methods (Fig. 5B) showed that the first two principal components accounted for 89.9% and 6.2% of the total variance, respectively. The cumulative contribution of PC1 and PC2 was 96.1%, which explained the vast majority of the variation in the dataset. Sample grouping was evident from the PCA plot, with the VTD samples located on the upper side of the x-axis and the CTD samples located on the lower side of the x-axis, indicating a significant (*p* < 0.05) difference in aroma compounds between the VTD and CTD-dried figs. The W3S sensor (alkanes and aliphatics) contributed the most to VT-E, while the W6S (hydrides) and W5S (nitrogen oxides) sensors contributed the most to the CT-E samples, while W5C (olefinic), and the W3C (aromatic molecule) sensors contributed the most to the FS data.

### 3.7 Electronic tongue analysis

Currently, the electronic tongue, as an instrumental method, has been widely applied in the qualitative and quantitative study of food flavor characteristics (Liao et al., 2024). The differences in taste response signals for fresh, VT-V, VT-E, and CT-E fig samples were relatively small (Fig. 5C), indicating similar taste component compositions across the samples. During the drying process, the astringency showed a gradual increase. The saltiness signal values for fresh figs and VT-V were − 9.13 and − 8.06, respectively, with no significant difference. However, the saltiness signal values for VT-E and CT-E increased to −3.35 and − 3.12, slightly higher than the human sensory threshold for saltiness (human sensory thresholds for sourness and saltiness are −13 and − 6, respectively (Xia et al., 2021)), suggesting that dried figs have a more complex sensor-derived taste profile. The sourness values for all samples were below the neutral point, indicating that the sourness was almost imperceptible throughout the drying process. The sweetness signal values significantly increased after drying, with the sweetness value for VT-E reaching a maximum of 10.35, consistent with the higher total sugar content in VT-E figs obtained in section 3.4.1.

To further analyze the impact of different drying treatments on the taste characteristics of figs, PCA was performed on the electronic tongue response values. As shown in Fig. 5D, the contribution rates of PC1 and PC2 were 89.9% and 8.5%, respectively, with a cumulative contribution of 98.4%, indicating that the methods had good feasibility (Li et al., 2019). Data points for different samples were distributed in four quadrants, suggesting significant differences in taste characteristics among the groups. Notably, the sample point for VT-V was relatively farther from the points for the other three samples, indicating that the taste characteristics changed significantly during the drying process. The sample points for VT-E and CT-E were relatively close, with sweetness contributing more to the recognition of VT-E, while sourness contributed more to the recognition of CT-E, suggesting that figs dried under variable temperature drying (VTD) conditions have a higher sweetness-related sensor signal.

### 3.8 Correlation analysis of volatile substances and non-volatile substances

**Fig. 6:**
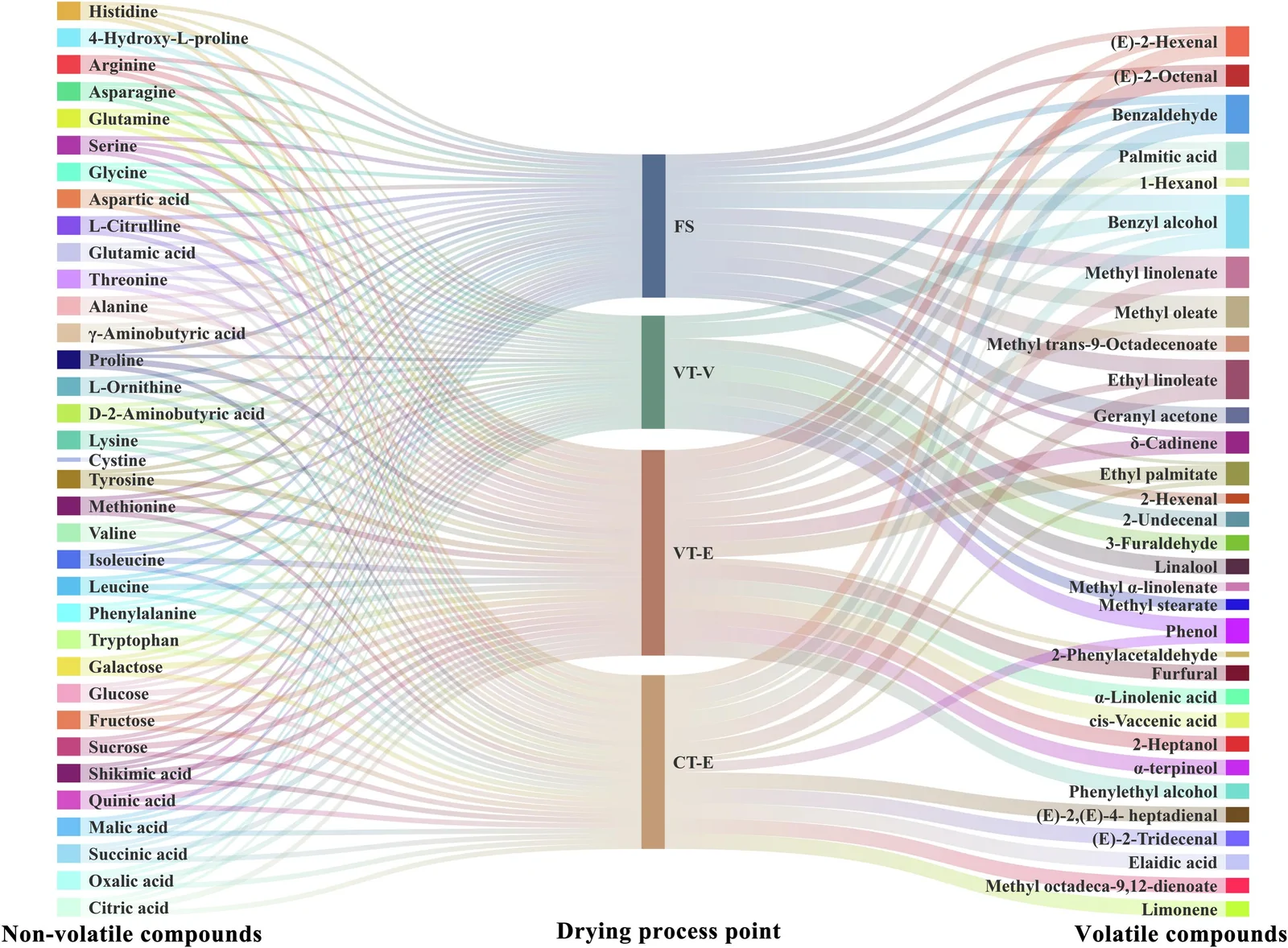
Sankey diagram of volatile and non-volatile compounds across drying process points in figs. FS, fresh sample; VT-V, variable temperature drying-variable temperature point sample; VT-E, variable temperature drying-end point sample; CT-E, constant temperature drying-end point sample. **Note:** The width of alluvial flows indicates the strength of compound-to-compound statistical associations. This figure only visualizes correlative patterns and does not confirm causal metabolic reactions or chemical transformations.

The Sankey diagram (Fig. 6) clearly shows the relationship among non-volatile precursors, different drying stages and volatile compounds, reflecting the potential connections established based on content changes or correlation analysis, and does not represent the direct chemical transformation path (Krumsiek et al., 2011). The CORNETO framework also emphasizes that the correlation method only captures statistical associations and does not provide causal interpretability (Rodriguez-Mier et al., 2025). Nevertheless, such correlation analyses can still provide valuable evidence for inferring the main reaction mechanisms.

Distinct association patterns were observed among the four stages. In FS, amino acids, sugars, and organic acids were broadly associated with benzyl alcohol, geranyl acetone, unsaturated fatty acid esters, and several aldehydes and alcohols, including (E)-2-hexenal, (E)-2-octenal, and 1-hexanol. This pattern may reflect the coexistence of endogenous metabolites and volatiles derived from enzymatic reactions or lipid oxidation in fresh tissues. The VT-V network contained fewer connections and was mainly associated with 2-hexenal, 2-undecenal, 3-furaldehyde, benzaldehyde, benzyl alcohol, linalool, and fatty acid esters.

The VT-E stage exhibited the largest number of associations, connecting non-volatile components with 16 tentatively assigned volatile compounds. Notable associations involved benzaldehyde, furfural, 2-phenylacetaldehyde, benzyl alcohol, 2-heptanol, (E)-2-hexenal, (E)-2-octenal, and fatty acid esters. These network features coincided with the marked increase in the total semi-quantitative volatile abundance in VT-E, which was driven primarily by benzaldehyde. Associations of amino acids and soluble sugars with aromatic aldehydes and furans are consistent with the possible involvement of amino-acid-related thermal reactions and carbohydrate degradation during the final 80 °C treatment (Huang et al., 2023; Zhao, Al-Dalali, et al., 2025; Zhao, Bian, et al., 2025; Zhao, Liu, et al., 2025). In CT-E, the network was mainly linked to (E)-2-hexenal, (E)-2-tridecenal, (E)-2, (E)-4-heptadienal, benzaldehyde, benzyl alcohol, limonene, and lipid-related compounds. Nevertheless, the lower total volatile abundance in CT-E indicates that the number or strength of correlations alone does not represent the amount of volatile compounds formed. Overall, the results suggest that the drying stage was associated with coordinated changes in non-volatile composition and the instrumental volatile profile. However, concentration effects, moisture loss, tissue disruption, and shared temperature responses may also contribute to these correlations; therefore, the proposed chemical relationships require validation using targeted precursor-tracing or controlled model-reaction experiments.

## 4 Conclusions

This study optimized a two-stage VTD process for Xinjiang figs using browning degree and specific energy consumption as the process-level responses. The optimal conditions were a pre-temperature of 55 °C, a post-temperature of 80 °C, and a loading capacity of 2.73 g/dm^2^. Compared with CTD at 60 °C, VTD shortened the drying time by 44.8%, reduced specific energy consumption by 20.4%, decreased the browning degree by 22.8%, and better maintained the porous microstructure of dried figs. VTD also resulted in 17.59%, 27.78%, and 43.29% higher retention of total phenolics, total flavonoids, and ascorbic acid, respectively, than CTD. These changes were accompanied by higher antioxidant capacity, although the possible contribution of thermally formed reducing compounds cannot be excluded.

The corrected GC–MS results showed that the summed semi-quantitative volatile abundances were 39.78 and 7.01 μg/g DW in VT-E and CT-E, respectively. This difference was mainly driven by benzaldehyde, which accounted for approximately 68% of the summed abundance in VT-E, rather than by a uniform increase in all volatile compounds. Changes in benzaldehyde, 2-phenylacetaldehyde, furfural, benzyl alcohol, sugars, and free amino acids were consistent with possible Maillard-type, Strecker-related, and sugar-degradation reactions during the final VTD stage. However, these pathways remain inferential because pathway-specific intermediates and reaction markers were not measured. The electronic-nose and electronic-tongue results further indicated treatment-dependent instrumental odor and taste patterns, but they should not be interpreted as evidence of sensory preference.

Overall, two-stage VTD improved drying efficiency and nutrient retention and produced a volatile profile distinct from that obtained by CTD at 60 °C under the tested conditions. However, the optimization was based only on browning degree and energy consumption, and the study was conducted at laboratory scale using a single fig cultivar without additional constant-temperature controls or human sensory evaluation. Therefore, validation across cultivars, sensory-panel testing, additional temperature controls, pilot-scale trials, and techno-economic analysis are required before industrial implementation.

## CRediT authorship contribution statement

**Yaxuan Liao:** Writing – review & editing, Writing – original draft. **Liqing Yang:** Writing – original draft, Investigation. **Hao Dong:** Formal analysis, Data curation. **Weida Zhang:** Validation, Investigation. **Lingling Li:** Validation, Formal analysis. **Minrui Guo:** Validation, Conceptualization. **Wanting Yang:** Validation, Supervision, Conceptualization. **Guogang Chen:** Supervision, Resources, Funding acquisition, Conceptualization.

## Declaration of competing interest

The authors declare that they have no known competing financial interests or personal relationships that could have appeared to influence the work reported in this paper.

## Data Availability

Data will be made available on request.
