# Supplementary material for: Effects of variable-temperature drying on nutritional components, antioxidant capacity, and flavor (volatile and non-volatile compounds) of Xinjiang figs
Source: Food Chem X. 2026 Sep 12;39:104439. doi: 10.1016/j.fochx.2026.104439 (PMC13594592; doi:10.1016/j.fochx.2026.104439)
Supplement: Supplementary file 1 [file mmc1.docx]

**Supplementary materials**

**Table S1** Response surface methodology design and experiment results

|  | Pre-temperature | Post-temperature | Loading capacity (g/dm^2^) | Browning degree | SEC (kWh/kg) |
| --- | --- | --- | --- | --- | --- |
| 1 | 60 | 80 | 3.5 | 0.937 | 5.67361 |
| 2 | 55 | 80 | 2.75 | 0.457 | 4.55972 |
| 3 | 60 | 85 | 2.75 | 1.019 | 5.30556 |
| 4 | 55 | 80 | 2.75 | 0.437 | 3.90278 |
| 5 | 55 | 80 | 2.75 | 0.494 | 3.75278 |
| 6 | 55 | 85 | 2 | 0.948 | 6.01389 |
| 7 | 55 | 85 | 3.5 | 0.895 | 5.33681 |
| 8 | 60 | 75 | 2.75 | 0.791 | 5.13876 |
| 9 | 50 | 75 | 2.75 | 0.558 | 5.03854 |
| 10 | 55 | 75 | 2 | 0.622 | 5.86254 |
| 11 | 55 | 75 | 3.5 | 0.840 | 5.31713 |
| 12 | 60 | 80 | 2 | 0.752 | 6.36806 |
| 13 | 50 | 85 | 2.75 | 0.771 | 4.92014 |
| 14 | 55 | 80 | 2.75 | 0.447 | 4.44722 |
| 15 | 55 | 80 | 2.75 | 0.450 | 4.42708 |
| 16 | 50 | 80 | 2 | 0.719 | 6.33102 |
| 17 | 50 | 80 | 3.5 | 0.698 | 5.60069 |

**Note:** SEC: specific energy consumption.

**Table S2** Analysis of variance for single indicator regression equation

| Indexes | Browning degree | | | | | SEC (kWh/kg) | | | | |
| --- | --- | --- | --- | --- | --- | --- | --- | --- | --- | --- |
| Source of variance | Sum of squares | Degree of freedom | Mean square | *F* value | *p* value | Sum of squares | Degree of freedom | Mean square | *F* value | *p* value |
| Model | 0.6100 | 9 | 0.0680 | 60.49 | < 0.0001 | 9.0700 | 9 | 1.0100 | 12.75 | 0.0014 |
| A | 0.0710 | 1 | 0.0710 | 63.38 | < 0.0001 | 0.0443 | 1 | 0.0443 | 0.56 | 0.4783 |
| B | 0.0840 | 1 | 0.0840 | 75.52 | < 0.0001 | 0.0060 | 1 | 0.0060 | 0.08 | 0.7906 |
| C | 0.0140 | 1 | 0.0140 | 12.10 | 0.0103 | 0.8760 | 1 | 0.8760 | 11.08 | 0.0126 |
| AB | 0.001 | 1 | 0.0010 | 0.05 | 0.8290 | 0.0203 | 1 | 0.0203 | 0.26 | 0.6276 |
| AC | 0.0110 | 1 | 0.0110 | 9.49 | 0.0178 | 0.0003 | 1 | 0.0003 | 0.01 | 0.9509 |
| BC | 0.0180 | 1 | 0.0180 | 16.42 | 0.0049 | 0.0043 | 1 | 0.0043 | 0.06 | 0.8216 |
| A^2^ | 0.0810 | 1 | 0.0081 | 72.74 | < 0.0001 | 1.6300 | 1 | 1.6300 | 20.59 | 0.0027 |
| B^2^ | 0.1500 | 1 | 0.1500 | 134.14 | < 0.0001 | 0.2869 | 1 | 0.2869 | 3.63 | 0.0985 |
| C^2^ | 0.1400 | 1 | 0.1400 | 122.67 | < 0.0001 | 5.6000 | 1 | 5.6000 | 70.87 | < 0.0001 |
| Residual | 0.0078 | 7 | 0.0011 |  |  | 0.5535 | 7 | 0.0791 |  |  |
| Lack of fit | 0.0059 | 3 | 0.0019 | 4.11 | 0.1029 | 0.0246 | 3 | 0.0082 | 0.06 | 0.9771 |
| Pure error | 0.0019 | 4 | 0.0048 |  |  | 0.5288 | 4 | 0.1322 |  |  |
| Total | 0.6200 | 16 |  |  |  | 9.6200 | 16 |  |  |  |
| Coefficient | R^2^ = 0.9873  R^2^_adj_ = 0.9710 | | | | | R^2^ = 0.9425  R^2^_adj_ = 0.8685 | | | | |

**Note:** *p* < 0.0001 indicates a highly significant difference. SEC, specific energy consumption

**Table S3** Effects of different drying methods on the color and texture characteristic of figs.

| Indicators | FS | VT-V | VT-E | CT-E |
| --- | --- | --- | --- | --- |
| *L* | 51.39 ± 1.57^a^ | 46.17 ± 1.03^b^ | 39.32 ± 1.21^c^ | 36.83 ± 0.82^d^ |
| *a* | 8.43 ± 0.56^b^ | 8.86 ± 0.70^b^ | 13.54 ± 1.32^a^ | 13.22 ± 0.96^a^ |
| *b* | 35.51 ± 1.83^a^ | 34.16 ± 1.55^a^ | 28.33 ± 1.38^b^ | 26.34 ± 1.04^c^ |
| *ΔE* | - | 7.38 ± 0.52^c^ | 17.95 ± 0.87^b^ | 22.61 ± 1.41^a^ |
| Hardness (g) | 314.03 ± 48.38^c^ | 417.27 ± 40.05^b^ | 407.91 ± 32.70^b^ | 569.91 ± 58.36^a^ |
| Adhesiveness (N·mm) | -24.40 ± 3.44^c^ | -16.51 ± 1.74^b^ | -1.96 ± 0.57^a^ | -1.17 ± 0.09^a^ |
| Gumminess (g) | 90.72 ± 4.63^c^ | 140.78 ± 2.08^b^ | 170.29 ± 7.55^a^ | 192.18 ± 14.06^a^ |
| Chewiness (g) | 60.49 ± 1.21^c^ | 78.12 ± 1.55^b^ | 108.68 ± 10.71^a^ | 120.03 ± 11.92^a^ |
| Springiness | 0.52 ± 0.08^b^ | 0.55 ± 0.15^b^ | 0.66 ± 0.07^a^ | 0.66 ± 0.10^a^ |
| Cohesiveness | 0.28 ± 0.01^c^ | 0.40 ± 001^b^ | 0.43 ± 0.01^ab^ | 0.45 ± 0.04^a^ |

**Note:** The different representations of small letters in the same index are significantly different (*p* < 0.05). FS, fresh sample; VT-V, variable temperature drying-variable temperature point sample; VT-E, variable temperature drying-end point sample; CT-E, constant temperature drying-end point sample.

**Figure S1.** Representative total ion chromatograms (TICs) of volatile compounds. (A) FS; (B) VT-V figs; (C) VT-E figs; (D) CT-E figs. X-axis represents retention time (min); Y-axis represents total ion intensity. Each chromatogram is selected from one representative biological replicate of the corresponding treatment. FS, fresh sample; VT-V, variable temperature drying-variable temperature point sample; VT-E, variable temperature drying-end point sample; CT-E, constant temperature drying-end point sample.
